# Personalization of the MES System to the Needs of Highly Variable Production

**DOI:** 10.3390/s20226484

**Published:** 2020-11-13

**Authors:** Bożena Zwolińska, Agnieszka Anna Tubis, Norbert Chamier-Gliszczyński, Mariusz Kostrzewski

**Affiliations:** 1 Faculty of Mechanical Engineering and Robotics, AGH University of Science and Technology, 30-059 Kraków, Poland; bzwol@agh.edu.pl; 2 Faculty of Mechanical Engineering, Wroclaw University of Science and Technology, 50-371 Wrocław, Poland; agnieszka.tubis@pwr.edu.pl; 3 Faculty of Mechanical Engineering, Koszalin University of Technology, 75-453 Koszalin, Poland; 4 Faculty of Transport, Warsaw University of Technology, 00-662 Warsaw, Poland; mariusz.kostrzewski@pw.edu.pl

**Keywords:** MES system, process variability, smart factory, performance indicators, Industry 4.0, Logistics 4.0

## Abstract

The new generation Manufacturing Executions System (MES) is considered as one of the most important solutions supporting the idea of Industry 4.0. This is confirmed by research conducted among companies interested in the implementation of the Industry 4.0 concept, as well as the publications of researchers who study this issue. However, if MES software is a link that connects the world of machines and business systems, it must take into account the specifics of the supported production systems. This is especially true in case of production systems with a high level of automation, which are characterised by flexibility and agility at the operational level. Therefore, personalization of the MES software is proposed for this class of production systems. The aim of the article is to present the MES system personalization method for a selected production system. The proposed approach uses the rules of Bayesian inference and the area of customisation is the technological structure of production, taking into account the required flexibility of the processes. As part of the developed approach, the variability index was proposed as a parameter evaluating the effectiveness of the production system. Then, the results of evaluation of the current system effectiveness by use of this index are presented. The authors also present the assumptions for the developed MES personalization algorithm. The algorithm uses the rules of Bayesian inference, which enable multiple adjustments of the model to the existing environmental conditions without the need to formulate a new description of reality. The application of the presented solution in a real facility allowed for determining production areas which are the determinants of system instability. The implementation of the developed algorithm enabled control of the generated variability in real time. The proposed approach to personalization of MES software for a selected class of production systems is the main novelty of the presented research and contributes to the development of the described area of research.

## 1. Introduction

Previous industrial revolutions were triggered by the emergence of a single breakthrough technology that changed the existing manufacturing system. Meanwhile, the fourth industrial revolution is defined by many authors as a combination of technologies available on the market from various fields [1]. These technologie0s are accompanied by simultaneous growth in the gathering and availability of data on the shop-floor, due to the current trend of using sensors and monitoring instrumentation [2]. For this reason, their use in an enterprise is often associated with the intensive implementation of information and communication technologies in the context of digitisation of industrial processes, cyber-physical systems (CPSs), Internet of Things (IoT), human–robot collaboration, and real-time big-data processing capabilities [3]. Additionally, the need to transfer huge quantities of information in a fast and reliable manner demands advanced communication methods, drawing extensive research interest in technologies that meet the increasingly stringent requirements of specific industrial applications [4].

In many countries around the world, and also in Poland, increasingly more investment focused on information technologies is being observed. Their use in manufacturing enterprises is now a priority. The implementation of advanced information technologies is an opportunity for the development of modern enterprises and their achievement of the desired competitive position on the market. For this reason, Polish producers are increasingly interested in the concept of Industry 4.0. This was confirmed by the research entitled “Readiness of production companies to implement Industry 4.0 solutions” [5], which was carried out by the executive search and development firm PSI Polska among 228 companies operating in four sectors: machinery and equipment, cars and transport equipment, furniture and metal products. According to these studies, as many as 70% of companies familiar with the Industry 4.0 concept have planned or have already started to implement solutions that are part of this concept. Large companies were placed in leading positions in that market analysis because such actions were taken by over three quarters of them (77%). Among medium-sized players, this indicator was equal to 59%, yet both groups were equally eager to implement these technologies in the near future. Manufacturers of machinery and equipment (87%), as well as cars and transport equipment (70%), were the most enthusiastic about the above-mentioned concept.

The growing interest of producers in the Industry 4.0 concept and the related expectations must be supported by appropriate information technologies (IT). Only the functionality of an appropriate IT system is able to meet the requirement of faster real-time response to information reported at the level of a single production station, regarding performance, downtime, product quality, etc. The system that meets the above expectations and responds to the needs of Industry 4.0 is the Manufacturing Executions System (MES). MES manages, monitors, and synchronises the execution of real-time, physical processes involved in transforming raw materials into intermediate and/or finished products. MES provides feedback on process performance, as well as support component- and material-level traceability, genealogy, and integration with process history, where required. The importance of using the MES in production processes implemented in accordance with the concept of Industry 4.0 is confirmed by the research described in [6]. The study “On the way to Economy 4.0” was conducted by the leading technology media, data and marketing service company IDG in partnership with ABB. The 108 companies participated in the survey, including representative companies of industry, production, and mining (40%), FMCG and trade (15%), telecommunications (10%), and others. According to the abovementioned study’s results, the implementation of the MES ranked first among the planned technologies related to the Economy 4.0 (as the concept integral to Industry 4.0). Additionally, the research presented in [7] proved that the new generation MES is considered as one of the most important solutions supporting the idea of Industry 4.0. For this reason, as many as 25% of respondents indicated this system as a planned implementation in the near future. One of the most important reasons why manufacturing companies invest solutions of MES class is the promise of generating large savings by decreasing machine downtime and failure, and effective monitoring of the working time of devices and personnel in particular production halls [7].

Almada-Lobo [8] defined four main pillars that MES system should contain to meet the requirements of Industry 4.0 [8]: decentralisation, vertical integration, connectivity and mobile, and cloud computing coupled with advanced analysis. MES among enterprise information systems (such as Enterprise Resource Planning—ERP, Supply Chain Management—SCM, Customer Relationship Management—CRM, etc.) plays a significant role in the daily on-time operation of modern enterprises [9]. As it was mentioned in [10,11], producing companies have relied heavily on manual feedback in MES in the past decade. Nevertheless, expansions of new technologies such as CPSs and IoT have allowed producing companies to collect data from multiple data sources through Radio Frequency Identification Devices (RFID), bar code, Manufacturing Data Collection (MDC), and other data acquisition systems, it was enumerated in [10,11].

Industry 4.0 is to integrate the real world (production machines) with the virtual world (information technology). As indicated above, the MES software should be the link between the world of machines and business systems. However, for this to be possible, the MES structure must reflect the specificity of the supported production system. In the case of non-mass production, which has high requirements for flexibility to adapt to changing demand, the standard MES solution will not meet the expectations related to the integration of the real and virtual world. Therefore, there is a need to personalize them to the specificity of production processes and machine systems. For this reason, the aim of the article is to present the MES system personalization method for a selected production system. The proposed approach uses the rules of Bayesian inference and the area of customisation is the technological structure of production, taking into account the required flexibility of the process.

The main contribution of the article to the area of research can be considered as:

- proposing the variability index as a parameter evaluating the effectiveness of the production system (in contrast to traditional performance indicators such as Overall Equipment Effectiveness—OEE, Overall Operations Effectiveness—OOE, Total Effective Equipment Performance—TEEP);
- presenting the results of the evaluation of the effectiveness of the current system based on the variability index;
- development of the MES software personalization algorithm, which takes into account the rules of Bayesian inference;
- verification of the functioning of the algorithm in the selected production system, including the possibility of predictive determination of the variability index values.

Therefore, Section 2 presents a brief review of the literature on smart factories in the context of the Industry 4.0 concept and the role of new MES systems in the operation and development of these factories. The articles cited above do not constitute a complete review of the literature in the studied area, but they have been selected in such a way as to define the specifics of the research area and the need for the conducted analyses. In Section 3, the applied research approach and assumptions for the proposed algorithm supporting the functioning of the personalized MES system are presented. Next, the specificity of the production system was described, which justifies the need to customise the system. Section 4 presents the most important results obtained in the process of the proposed approach validation in an actual production system. The last part of the article (Section 5) discusses the significant benefits obtained in connection with the implementation of the proposed solution in the enterprise, as well as the barriers encountered in the implementation process.

## 2. Literature Review

In the current section, a narrative literature review is presented. This literature review is enriched with two research questions (RQ: RQ1, RQ2). The first RQ1 is namely: in which research areas are the contributions on MES applications listed? And the second RQ2 is: what sources, scholars and papers have been most influential in research on using MES in aircraft production? The answers are given at the second half of this section.

In [12] it was mentioned that MES were introduced in the mid-1990s (whereas, in [13] (p. 13) the author mentions that the origins of the MES concept were to be found in the early 1980s). In the paper, the authors identified, investigated, and reviewed the major issues connected to MESs back then. As the authors of literature review paper [14] mentioned, Industry 4.0 solutions has a significant influence on the development of MESs. This statement resulted as a consequence of their recently published revision of standards, ontology-based methodologies, and tools dedicated to development of MESs. The authors attempted to predict the trends that determine the expansion of the next MES generation, which might encompass especially that manufacturing operations generate enormous data quantities (industrial big data) [15]. The connection between the pairing of Industry 4.0 and MES is underscored by [8,16,17] as well. The author of [8] briefly discussed the contradictions and analysed the MES providers’ reactions to the impact of Industry 4.0 for cessation of centralised systems’ existence (decentralisation in a logical way rather than a physical one was taken into consideration). The evolution of the MESs was presented in review part of the paper [16], whereas in the review paper [17] the opportunities for further development of MESs resulting from the recent developments of Industry 4.0 were analysed and presented. The challenges associated with MESs functionality adjustment in the light of Industry 4.0, as technologically heterogenous systems in comparison to the other manufacturing systems were analysed in [18].

Manufacturing systems, including MES, were reviewed in [19] and a comprehensive overview of the actual MES solutions with special attention to MES-complementary systems and tools was presented in [20]. Vertical integration of factory automation systems into MESs was presented in [21]. It was described as a technical area of interest in the overview of actual technologies and trends in factory automation (smart factory), therefore in connection to Industry 4.0 as well.

Industry 4.0 is a combination of information technology and highly controllable computer-driven machines [22]. In the manufacturing area, the Industry 4.0 vision has promoted smart manufacturing and smart factories concepts by augmenting all assets with sensor-based connectivity [23]. Smart manufacturing improves long-term competitiveness by optimising labour, energy, and material to produce a high-quality product, and finding a rapid response for variation in market demands and delivery time [24]. For this reason, in the smart factory the system should be context-aware and should support particular companies’ employees and machines executing their tasks based on information from both physical and virtual environments [25]. Numerous studies have been dedicated to initiating smart factories, investigating their features, designing their key enablers, and implementing them on the ground [26]. The example of one such study are the results described in [27]. The authors developed a framework in order to support the identification and description of a smart factory system. They suggest requirements that the factory should fulfil to increase its smartness. The following principles were developed for smart factories design in [28]: Interoperability, Virtualisation, Decentralisation, Real-Time Capability, Service Orientation, and Modularity. These principles are intended to assist companies in the identification of potential Industry 4.0 pilots that can then be implemented. Research on the future development of smart factories also merits attention. It is expected that smart factories will evolve into self-adaptive factories where all the elements will be interconnected, exchanging information, recognising and assessing situations, and organically fusing the physical world with the cyber world [29,30], especially that CPS analyses actual data collected in real-time by merging information systems, including the IoT, MES, and advanced planning and scheduling systems (APS) [31]. More specific research connected to use of the MES are mentioned below. 

CPS given in [31] were described with their core elements where among sub-systems the following can be found: Key Performance Indicator (KPI), Simulation Detection and Coordination, and Big Data Analytics sub-systems. The last sub-system was developed to store big data generated from IoT/sensor and MES. In the authors’ framework, the data collected with the use of IoT and MES allows to predict metal-casting defects, whereas APS created new schedules by reflecting irregularities (as mentioned defects) and sent them to the MES and particular coordinator.

In [32] the authors described a modern shipyard which applied actual technologies according to the Industry 4.0 principles. The authors presented a fog computing based MES deployed in parallel with CPS that was developed for use of active RFID technology to track pipes which are essential in shipbuilding. The concept was developed in order to facilitate daily-basis operations at a pipe workshop. As the authors mentioned, MES is usually integrated with two additional software types: ERP and Product Life-cycle Management (PLM) software. Such a combination of software provides data sharing and smooth coordination between various functional areas, such as engineering, workshops, and the front office.

Research confirms that in order to build the smart factory, enterprises should improve their production systems, enhance controllability of production process, and reduce manual intervention in the workshop [33]. Based on the analysis of production data, smart factories can realise flexible manufacturing, dynamic reconfiguration, and production optimisation. Thanks to this, it is possible to quickly adapt the production system to changes in the business model and consumer shopping behaviour [34]. The reconfigurable production line is now a particularly desirable feature of smart factories. Many manufacturers today are forced to produce products in small batches, in many varieties; for this purpose, the production line should reconfigure its processes and recombine manufacturing units dynamically. Thanks to this, the company can adjust the type of product and production capacity in time actual to the existing needs [33]. An example of such a solution is the optimisation project leading to 2-stage production line reconfiguration, as described in [35]. In the first stage, a multiple-objective particle swarm algorithm was proposed to optimise cost, machine utilisation, operational capability and configuration convertibility. At the next stage, a maximum deviation method was used to choose an optimal scheme from the alternatives in order to avoid both subjectivity and uncertainty in the decision-making process.

‘Smart factory’ is the fundamental concept of Industry 4.0 and to commission it the field of ‘business information systems engineering’ (that includes innovative MES approaches) will become a key issue in its implementation [36]. This is due to the concept of Industry 4.0 expecting productivity improvement through fulfillment of the growing customer demands for faster real-time response via decentralised production control. These expectations can be fulfilled by MES to improve performance, quality, and agility for globalised manufacturing businesses [8]. This is also confirmed by the results of the research described in Matravadi and Møller [16], whose aim was to find a connection between the MES tool and Industry 4.0 to highlight its importance for future smart factories. However, in order for MES to play a greater role in smart factories than just providing features for manufacturing management, it must be expanded to the requirements of the new generation [37]. The new class of MES is expected to provide further real-time information to the operational departments by providing an ‘all-round view’ of all resources involved in the production, and will act as a manufacturing cockpit [16] (MES cockpits i.e., “dashboards for manufacturing operations management used by production supervisors on the manufacturing control level”, the term which was inspired by dashboards in automobiles and aircrafts [13,38,39]). Additionally, modern cloud based MES has to deal with product traceability in distributed manufacturing, for example “where workflows of multiple factories are coordinated centrally to provide plant managers with real-time tracking, visibility, and control across several plants” [40]. With data collection, analysis, and communication functions across the value chains, new generation MES will serve as a platform for implementation of the Industry 4.0 technologies and realising this opportunity [17,41].

The authors of [42] summarised their research framework on traceability systems for production with the conclusion that traditional traceability systems such as MES increase costs when combined with the increase in logistics complexity, therefore indispensable investments may be hardly acceptable for small companies [43].

The authors of [44] presented the RFID-enabled real-time manufacturing execution system which was developed for manufactures of large-scale and heavy-duty machineries. In [45] the researchers tackled the problem of inefficient wireless data acquisition in the shop-floor environment by adopting RFID technology to capture signals from movable objects (their positions) in continuous manner. The authors analysed various positioning algorithms and their experiments were conducted in a university’s laboratory.

The above-mentioned references have allowed to answer the first RQ1 which is: in which research areas are the contributions on MES applications listed? These areas are—to mention only the selected ones—engine construction [31], ship construction [32], heavy-duty machinery [44], metal-mechanic industry [46], and university research [45]. In general, it can be mentioned that these contributions, as in the case of the whole Industry 4.0, are located in the following types of industries: aerospace, automobile, iron and steel and petrochemical [47]. More recently, research has entered the next stage of development i.e., autonomous intelligent vehicles are combined with MES in order to build an even more up-to-date and significant—from the Industry 4.0 perspective—automated and interconnected manufacturing environment [48].

As it was suggested by RQ2 and it is mentioned further in the paper, a part of its goals are connected to fuel system components manufacturing for various types of aircraft. Therefore, the application of MES in research linked to such an industry are worth analysing. The four following paragraphs include answers to RQ2, which is: what sources, scholars and papers have been most influential in research on using MES in aircraft production?

The authors of [49] presented the challenges of MES implementation in China. As far as the aircraft companies are concerned, the authors mentioned with reference to [50] that for successful MES application pre-reengineering work has to include basic data standardisation and redefined position/roles. On the other hand, the authors reported, based on [51], that a particular Chinese aircraft tools manufacturer found the shopfloor always in overload state and in-process inventory was always high without MES.

In [52], the author described MES implementation in company aircraft production line. The MES software couple with plenty other systems was applied in order to support the F-35 production line. At the same time next generation MES was announced in this paper to expand business benefits.

The authors of [11] presented an industrial application connected to an aero-engine blade manufacturing factory which produces different kinds of aero-engines for jet fighter, transport aircraft and passenger planes. The authors found a certain problem of bottleneck through the rough-cut capability planning in the MES while using a 4-axis CNC milling machine. Therefore, the authors took into consideration this bottleneck machine scheduling as the key issue. Previously, in [53] use of a hybrid genetic algorithm for operation scheduling was presented, which was integrated by the researchers in MES for multi-objective scheduling.

The authors of [54] presented a case study of an aircraft components and assemblies’ manufacturer. The company’s activities are controlled and monitored through MES, as well as production environment directives communicated through MES. Among this system’s responsibilities the following can be mentioned: facilitation of buffer management [55], work-in-progress control and prioritisation on a production control level. In this paper, the authors deliberated various factors which can be characterised by the negative impact on MES in facilitation of recovery.

In [56], the researchers presented a project generating features data by implementation of quality control for the production of carbon fibre components of aircraft. In order to support the decision-making process connected to particular features, an extensible hybrid decision support system was proposed. This system combined software for 3D-based process visualisation of specific feature data supporting the execution of rework decisions and a web-based business analytics cockpit. The cockpit presented in a visual way the data generated by a simulation modelling tool (Plant Simulation) for various rework strategies, as well as data from MES or Production Data Acquisition (PDA) (both treated as subsystems providing helpful hints for the production’s optimisation). Carbon fibre reinforcing plastics for aircraft parts manufacturing together with the application of MES was also presented in [57] as a work-in-progress.

After the analysis of various references, the authors observed that the approach which uses the rules of Bayesian inference and the area of customization is the technological structure of production are hardly mentioned, in particular for aircraft items manufacturing. This research gap convinced the authors that the aim of the article should focus on the presentation of the MES system personalization method for a selected production system. This method includes the variability index as a parameter predesigned for evaluation of production system’s effectiveness and provides evaluation of effectiveness of a particular system based on the variability index. The results presented in the current article, connected to the evaluation of the effectiveness of the actual system, are based on this index.

## 3. Materials and Methodology

The results of the research presented in the article concern the class of production systems that are characterised by flexibility and agility at the operational level. Such a system enables manufacturing of a wide range of different products. It is the system with a high level of process automation that significantly reduces human participation in the production processes. The human work is limited to the correct setting of the machine parameters and ongoing process control and metrological measurements. For this reason, the described systems represent a significant potential for implementing solutions based on the fourth industrial revolution.

The tests were carried out in the production system in which fuel system components for various types of aircraft are manufactured. Production in the aviation sector is characterised by a high level of process and product quality. The restrictive quality requirements assume the small range of the manufacturing tolerances given that the manufactured products determine the safety of future users of the final product. For this reason, in the aviation sector, a key factor at all stages of production is the compliance with the highest safety requirements. The multifaceted quality of design, production, inspections, and repairs is defined by the AS/EN 9100 family of standards. The aviation industry is also characterised by the high requirements for the used solutions and materials. The input material applied for production is the austenitic nickel-chromium-based alloy (so-called Inconel). The waste from the input material in the form of chips from the machining processes accounts for between 60% and 80%. The rate of productivity defined by the ratio of the mass of the input material to the mass of the manufactured intermediate product is relatively low. This level results from the technology used and the established quality requirements.

The main advantage of the production system under consideration is the precision of the processes and the flexibility to adapt to customers’ expectations. These two factors should be constantly monitored and optimised. This approach differs significantly from the high volume and mass production systems. In both of these systems, the most common is a linear production structure in which the main parameters for assessing the effectiveness of the system are efficiency and productivity. Meanwhile, in the analysed production system there is a technological manufacturing structure, assortment diversification, niche demand for manufactured products and very restrictive quality requirements. These elements enforce control over the variability of the system operation states. The factor influencing this variability is primarily the diversified planning route resulting from the lack of repeatability of the technological route for short-series (single) orders. Each production order is treated individually and on a one-off basis, in accordance with DTO (design to order) and ETO (engineering to order).

All of the above factors affect the multifaceted absorption of the company’s resources: time, finances, material, and energy. In order to maximise the effectiveness of the entire enterprise system, multi-criteria optimisation of the production processes is necessary. The developed optimisation algorithms should also take into account the dynamic variability of the system operation states. In order to obtain the highest level of fit, the model layout should be updated with current data in real time. For this reason, it has been proposed to use algorithms with the application of Bayesian inference rules. These algorithms enable multiple adjustments of the model to the existing environmental conditions without the need to formulate a new description of reality. Bayesian inference is the method that allows for the dynamic calibration of the system’s operating parameters by modifying the prior distribution. The sampling frequency of the system operation states, which can also be the frequency of updating the prior distribution, depends on the dynamics of changes in the modelled system.

The control over the course of the production process is gained by using PLC drivers, industrial software such as MES, SCADA—Supervisory Control And Data Acquisition and ERP [58]. MES—among enterprise information systems e.g., ERP, SCM, CRM, etc.—plays a significant role in the daily on-time operation of modern enterprises [9]. These solutions contribute to the full transparency of the implementation of manufacturing processes. They also provide the required traceability, understood as the ability to reproduce the movement of objects and their surroundings [59]. However, it should be remembered that having a large amount of information does not mean that it is not used optimally. The maximum use of Industry 4.0 capabilities in the unit production with very restrictive quality requirements forces conscious and individual development and implementation of compatible models of the real and virtual world. Software solutions available on the market allow access to data and machines from anywhere in the world, allowing managers to have real-time control over the production system. Currently, this is no longer sufficient. In the virtualisation of real production systems, the current development trends are prediction and cybersecurity. Due to the increasing scale and complexity of cyberattacks, the control of the production modules is closed at the SCADA system level. The MES is often mistakenly perceived with the SCADA system. However, the latter is used to simplify the visualisation of information gathered about the production system. The MES-class systems are designed in such a way that the values of the system efficiency evaluation indicators are as up to date as possible at a given measurement moment. The implanted algorithms use real-time data. Thanks to the early response to potential changes or disturbances, it is possible to obtain maximum results of work.

**Figure 1:**
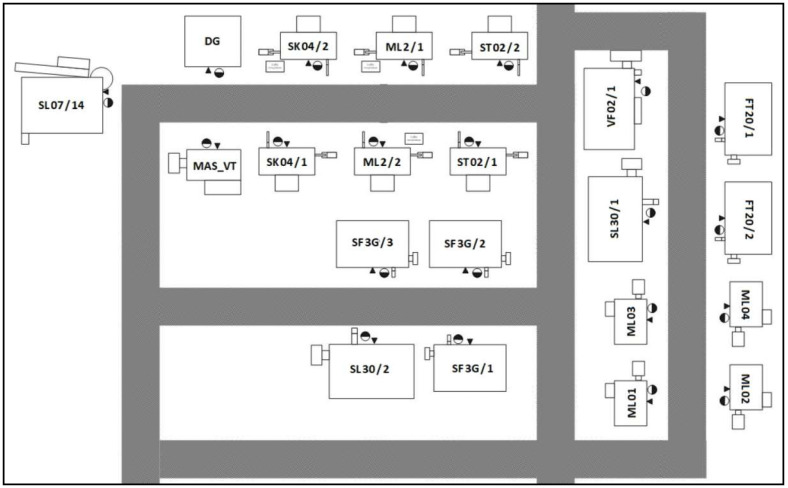
The machining department.

**Figure 2:**
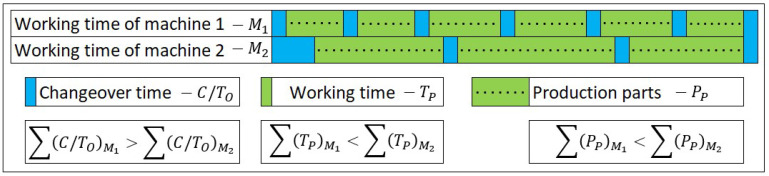
Workload graph for a variable production schedule.

The article presents the concept of the implementation of the MES software, which was personalised to the requirements of the analysed production system (Figure 1). The main area of personalisation is process flexibility and its technological production structure. The case described in the article is a reformatory one, because the main indicator of the effectiveness evaluation is the system variability. In the analyses performed to date, one of the basic parameters of the operational level assessment was the OEE indicator. Then, OOE and TEEP were used in order to obtain a more reliable assessment of the actual use of the potential of the machine park. The OEE indicator is the most frequently used indicator for assessing the effectiveness of the machine park usage. It is applied mainly in the automotive sector. However, for production systems with a large number of changeovers between short, unique production runs, OEE is unreliable; this is due to the difficulty of determining the values of standard task completion times including, in particular, the changeover times between individual products. Figure 2 presents a graphical interpretation of the machine load for two different production schedules: (a) short production runs with a relatively large number of changeovers; (b) relatively long production runs with a small number of changeovers in the assumed Δ*t*. There is a ‘learning’ effect for repeatable production (both in retooling processes as well as in task execution processes). This determines longer execution times of processes when they are performed the first few times. For the considered production system, the key performance indicator (KPI) is the quality of execution. Hence, technologically defined times have a tolerance margin, so that the time pressure does not reduce the assumed quality level. With such defined work standards, exceeding the assumed times of task completion occurs in emergency situations. As a result, it causes relatively high values of OEE indicators (see data presentation later in the article).

Performance (*P*) and quality (*Q*) parameters are determined identically for all three traditional indicators. In relation to OEE, availability is defined differently for the OOE and TEEP indicators (*A*). In the case of OEE, parameter *A* defines the so-called planned availability *A_s_* (Equation (1)). Then, *A_S_* is determined by the ratio of the actual production time to the production time minus any planned downtime (e.g., machine repairs and others). In the case of OOE, parameter *A* determines the operational availability of *A_O_* (Equation (2)). Then *A_O_* is determined as the ratio of the actual production time to the operational time, which is only reduced by independent breaks resulting, for example, from the lack of orders. However, in the TEEP indicator, the so-called total availability, *A_T_* (Equation (3)), which is defined by the ratio of the actual production time to the total operating time.

(1) $$A_{S}=\frac{t_{e}}{t_{o}-t_{p}},$$

(2) $$A_{O}=\frac{t_{e}}{t_{o}},$$

(3) $$A_{T}=\frac{t_{e}}{t_{a}},$$

where: $t_{e}$—effective time, $t_{p}$—planning standing time for changeover time or idle time, $t_{o}$—operation time (for 1 shift: $t_{o}=480\;\left[\min\right]$; for 2 shifts: $t_{o}=2\times 480\;\left[\min\right]$), $t_{a}$—machine access time ($t_{a}=24\times 60\;\left[\min\right]$). 

**Figure 3:**
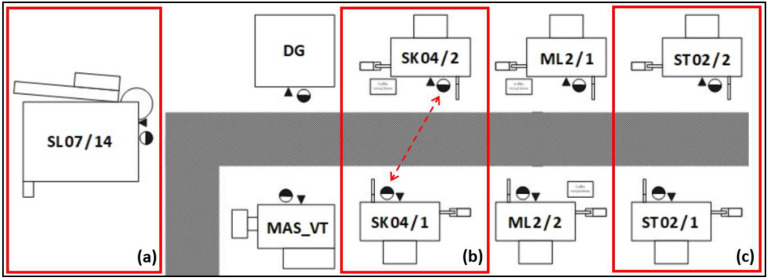
Diagram of the analysed production cells: (**a**) 1 operator + 1 machine; (**b**) 1 operator + 2 machines; (**c**) 2 operators + 2 machines.

In each of the traditional indicators, a certain modification was made to fit the specificity of the assessed system. However, these modifications were not sufficient for the class of production systems discussed in the article. For this reason, it turned out to be necessary to adjust the previously used work efficiency assessment indicators to the individual features of the analysed system. From the entire technological structure of the production system, production stands equipped with modern subtractive processing centres were separated. The diagram of the separate production system in which the validation of the developed MES class system was performed is shown in Figure 3.

All objects (machines) in the subtractive department are CNC machine parks with a tool depot with a capacity of 40 to 120 pieces. The company’s investment policy is the systematic purchase of modern equipment from various manufacturers. Diversification of suppliers (manufacturers) of machinery equipment results from the individual characteristics of the machine, e.g., inertia, which directly affects the quality of the product. For this reason, the objects (machines) shown in Figure 1 appear in the department no more than two of the same type.

The implementation project of the developed MES software started with the implementation on selected production cells in one of the separated sockets of the technological structure. Three different positions (shown in Figure 3) were subjected to a detailed analysis of the effectiveness of the innovative approach:

1. Production cell consisting of one machine and one operator.
2. Multi-machine production cell (2 machines) operated by one operator.
3. Multi-machine production cell (2 machines) operated by two operators.

The first stage of the system assessment was to analyse the continuity of material flow for the previously separated product families. The classification criterion determining belonging to the given family was the similarity of the technological route and the time of realisation of the processing stages. Analyses of OEE, OOE, and TEEP indicators were performed for the full year. In order to illustrate the changes in the values of the OEE, OOE, and TEEP indicators, one week was adopted as the billing cycle. In economic and financial balances, the system is assessed with the average values obtained at the turn of one month. However, in the area of the article, Δ*t* was assumed to be equal to one week in the period from 1 January to 31 December 2019. Then, on the basis of the analyses, it was decided that the simplest and the best parameter for assessing system variability will be the time of the whole process or its elementary part.

The paper [60] presents the author’s algorithm for assessing the variability that occurs in a flexible production system. The verification of the presented algorithm was carried out on the basis of data collected in the analysed generating system. The result of the research described in [60] was the determination of the existing correlations between the change in the time of task completion in individual processes (or their elementary stages) and the variability of the entire production system. This article stated that: The volatility index V is directly proportional dependent on the number of processing steps because the standard deviation as the root of the sum of variances of independent random variables with Erlang distributions is determined by the sum of the number of k elements performed at all processing steps [60]. The continuation of works related to the limitation of the existing variability resulted from the necessity to stabilise the system; this stabilisation will improve the continuity of material flow and eliminate blockages in the control process in the Quality Control Department. The manufactured semi-finished product is checked for compliance of performance after many elementary stages of the entire process due to the high quality restrictions. The input material (Inconel) used for production is an expensive raw material. Before starting the planned (ordered) production batch, first one product is manufactured in order to eliminate the costs of manufacturing products not compliant in terms of quality, then it is subjected to a detailed inspection, and after approval of the performance compliance the production of the remaining items is started. The final product inspection and approval process is carried out in the Quality Control Department (QCD), which is also responsible for collecting and archiving data on all products manufactured in the company. However, the tasks performed by the Quality Control Department are often a bottleneck at the operational level, due to the stringent flight safety requirements that apply to the company’s customers. At the same time, the specificity of production makes it impossible to reorganise the activities that are performed in the area of the QCD. In order to streamline the work of the entire production structure, a decision was made to adjust the operating level to the work cycle of the QCD. For this reason, it was decided to apply the variability assessment methodology presented in the book [61] along with the implementation of the stabilisation algorithm based on the rules of Bayesian inference.

**Figure 4:**
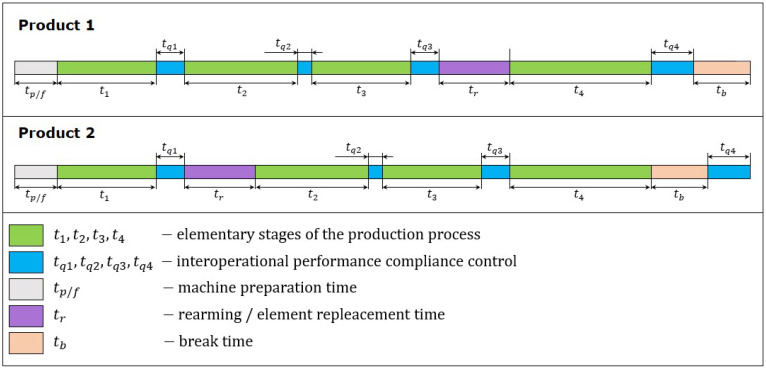
Differences in the execution of two identical products.

The machines used in the production are machine parks that can mount up to 40 different cutting tools. An elementary part of the process is defined as a component part of the technological sequence, e.g., face turning, which occurs between subsequent tool changes. The analysis of such fragmentation is forced by the current quality monitoring of the process implementation after its elementary part. Machined Inconel is a hard material, which results in faster wear of the cutting tools. Many studies have shown that even a few percent tool wear determines deviations in the tolerance of over ten percent [61]. In the case of the aviation industry, the lack of process stability, defined as the occurrence of measurement deviations, is unacceptable. The process of making the element is designed in such a way that, after each elementary stage of the technological process, the operator assesses the performance and makes decisions about the possible replacement of the tool. Then, the downtime is assigned to the time allocated for inspection or to the time of changeover, depending on the operator’s decision. Therefore, the implementation of two identical products will differ in the accumulated time of task completion and their sequence. An example of such differentiation is shown in Figure 4.

The variability in the proposed MES software algorithm was assessed in relation to the differences in the times of the individual stages. The conducted analyses proved that the range of time devoted to elementary tasks will be an appropriate parameter for the assessment of variability [62]. Therefore, the classic volatility index was used, expressed as the ratio of the standard deviation value to the mean value. The aim of the research was to develop a methodology of systemic (comprehensive) stabilisation of a system composed of many objects. For this reason, the method presented in the article by Zwolińska [60] was used in the variability assessment algorithm. The variability analysis was performed according to the volatility index *V*. The index of relative variation is a classic measure of the diversity of a feature distribution. The general form of the volatility index is defined as the ratio of the standard deviation to the expected value. 

In article [60], the impact of changes in the implementation times of any process from the planning (or technological) route on the occurring variability of the superior and subordinate systems was assessed. In order to establish the main areas of the impact of changes on the system, a certain modification was made in determining the value of variability. For this reason, the variability assessment was carried out for two variants:

(4) $$V_{1}=\frac{standard\;deviation\;of\;the\;superior\;system}{expected\;value\;of\;the\;subordinate\;system},$$

(5) $$V_{2}=\frac{standard\;deviation\;of\;the\;subordinate\;system}{expected\;value\;of\;the\;superior\;system},$$

According to the theory of complex systems formalised by Mesarovic [63], each complex system can be decomposed into a set of subsystems. Then the main system defined as the superior system is a set of separated subsystems (subordinate systems) connected by strictly defined relations. The article uses the analysis of abduction inference. First, a two-stage decomposition of the production system into a set of separate subsystems (divisions) was performed through induction. Next, another decomposition into a set of interdependent machines was made for the separated subsystem (Department of Mining Machining). In the second stage of abductive reasoning, deduction was used. The influence of the subordinate system (a single machine) on the working condition of the superior system (the Mining Machining Department) was analysed. For this reason, in further analyses, the superior system, system S, is the Mining Department together with the QCD, and the subordinate system is a set of separate machines $M_{j}$ dla j=1,2,…,J. These machines were analysed later in the article. 

For the above formulas, tests were carried out to determine the impact of changes in the execution time of a single process or its elementary part. It was found that due to the mutually exclusive dependencies of the parameter *λ_i_* and the number of *k_i_* elements performed in individual processes, it is not possible to obtain the values of $V_{1}$ and $V_{2}$ simultaneously converging to zero [60]. As a result of the research, it was found that: Limiting the variability of the entire production structure should be achieved by adjusting similar values of the standard deviation of the slave system to the expected value of the master system, regardless of the number of necessary production steps [60].

Knowing that there is a close correlation between $V_{1}$ and $V_{2}$ (if $V_{1}$ increases then $V_{2}$ decreases—and vice versa; if $V_{1}$ decreases $V_{2}$ automatically increases) in further studies to stabilise the system, one of the above relationships can be used. Therefore, in the proprietary MES system is implemented the relationship defined by the Formula (6):

(6) $$V\left(T_{M_{j}}^{S}\right)=\frac{D\left(T_{S}\right)}{E\left(T_{M_{j}}\right)},$$

where: $D\;\left(T_{S}\right)$—standard deviation of the random variable $T_{S}$ of the cumulative lead times of the production tasks in period Δ*t* performed for a set of machines: SL 07/14, SK 04/1, SK 07/2, ST 02/1, ST 02/2, $E\;(T_{M_{j}})$—expected value of a random variable $T_{M_{j}}$ of the accumulated time realized in period Δ*t* on one of the machines belonging to the set: SL 07/14, SK 04/1, SK 07/2, ST 02/1, ST 02/2.

By denoting by $\Delta V\left(T_{M_{j}}^{S}\right)$ the acceptable tolerance field of the variation coefficient values for the approved production schedule, the lower $V_{min}\left(T_{M_{j}}^{S}\right)$ and the upper $V_{max}\left(T_{M_{j}}^{S}\right)$ limit of the $V\left(T_{M_{j}}^{S}\right)$. Then:

(7) $$\Delta V\left(T_{M_{j}}^{S}\right)=V_{max}\left(T_{M_{j}}^{S}\right)-V_{min}\left(T_{M_{j}}^{S}\right)$$

For static systems in which there is no variability of WIP (Work-in-Progress) levels resulting from the variability of the planning route, there is a relationship:

(8) $$\forall \;i=1,2,\ldots ,\;n;\;{\left(V_{min}\left(T_{M_{j}}^{S}\right)\right)}_{i}=constans\;\wedge \;{\left(V_{max}\left(T_{M_{j}}^{S}\right)\right)}_{i}=constans\;$$

Then $\forall \;\Delta t_{i}=t_{i}-t_{i-1}$ the band of acceptable variability $\Delta V\left(T_{M_{j}}^{S}\right)$ is constant over time. We encounter such a situation in the balanced lines of the subject structure, e.g., the assembly line of the automotive sector. 

The production systems with a technological structure are characterized by the variability of the planning route. Then the field of tolerance of the variation index value deviation should be determined by the dynamic levels of $V_{min}\left(T_{M_{j}}^{S}\right)$ and $V_{max}\left(T_{M_{j}}^{S}\right)$. For the strictly defined S superior system and $M_{j}$ subordinate system (dla =1,2,…,J), the following symbols have been adopted: $V_{min}\left(T_{M_{j}}^{S}\right)=V_{min}$ and $V_{max}\left(T_{M_{j}}^{S}\right)=V_{max}$. Then:

(9) $$\forall \;i=1,2,\ldots ,\;n;\;{\left(V_{min}\right)}_{i}=V_{min}\left(i,k\right)\;\wedge \;{\left(V_{max}\right)}_{i}=V_{max}\left(i,k\right)$$

where: $V_{min}\left(i,k\right)$ and $V_{max}\left(i,k\right)$—these are functions of two variables depending on the period i and the cumulative number of manufactured products k in the period i. The value of k is a random variable that determines the number of products required to be made according to customer demand. 

For the variability defined by the Formula (6) for j=1,2,…,J, $E\left(T_{M_{j}}\right)$ changes at a certain fixed value of $D\left(T_{S}\right)$. Therefore, the levels of the lower $V_{min}$ and upper $V_{max}$ of the control line are relative to changes in the denominator:

(10) $$V_{min}=\frac{D\left(T_{S}\right)}{3Q}$$

(11) $$V_{max}=\frac{D\left(T_{S}\right)}{1Q}$$

where: 3Q and 1Q are respectively the third and the first quartile of the expected value $E\left(T_{M_{j}}\right)$ from the ordered set of permutations of the expected values of all considered objects of the master system. 

The first stage of applying the proposed concept of the variability assessment is the implementation of the developed algorithm in a complex production structure with a technological nature of production. In the first phase of implementing the mathematical model in the real system, the tolerance area $\Delta V\left(T_{M_{j}}^{S}\right)$ was determined as exactly 50% of the values of all $V\left(T_{M_{j}}^{S}\right)$ for j=1,2,…,J. In the next stages of the research, it is planned to verify the developed model, taking into account the quantile values. 

The model for determining the volatility and acceptable tolerance field outlined above includes five steps:

Step 1: determining the value $V\left(T_{M_{j}}^{S}\right)$ according to the Formula (4) in relation to the expected value of each object accepted for consideration $M_{j}$ dla j=1,2,…,J.
Step 2: sort the values of the indicator $V\left(T_{M_{j}}^{S}\right)$ by the non-increasing expected values of the set: $\left\{E\left(T_{M_{1}}\right),E\left(T_{M_{2}}\right),\ldots ,E\left(T_{M_{J}}\right)\right\}$.
Step 3: Defining Π permutation for the set order $\left\{E\left(T_{M_{j}}\right)\right\}$ for j=1,2,…,J.

where there is dependence:

(12) $$E\left(T_{\Pi M_{1}}\right)\geq E\left(T_{\Pi M_{2}}\right)\geq \ldots \geq E\left(T_{\Pi M_{J}}\right)$$

from which it follows:

(13) $$V\left(T_{\Pi M_{1}}^{S}\right)\leq V\left(T_{\Pi M_{2}}^{S}\right)\leq \ldots \leq V\left(T_{\Pi M_{J}}^{S}\right)$$

Step 4: Determination of the lower $V_{min}$ and upper $V_{max}$ of the control line for the third 3Q and the first 1Q quartile of values from the set of permutations of the set $\left\{E\left(T_{M_{j}}\right)\right\}$, according to the Formulas (10) and (11).
Step 5: modifying the production schedule so that each $V\left(T_{M_{j}}^{S}\right)$ for j=1,2,…,J is in the tolerance field $\Delta V\left(T_{M_{j}}^{S}\right)=V_{max}-V_{min}$.

The case described in the article is characterised by various variability. It results from the seasonality of demand, the assortment diversity of the reported demand, implemented technical and technological solutions and the internal organization of the production structure. For this reason, the analyses of the operating level show a relatively high dynamics of changes in the operating states of the system. Therefore, it was decided to use the rules of Bayesian inference, which enable multiple adjustments of the production system assessment parameters to the existing environmental conditions without the need to formulate a new algorithm each time. 

Manufacturing systems are characterised by a high degree of system complexity. The processes of effective improvement of any production area should take into account a comprehensive approach. The presented algorithm for stabilizing the operational level variability takes into account that one of the main causes of variability, defined by the formula $V\left(T_{M_{j}}^{S}\right),$ is the demand that varies over time. The changes in demand result from the assortment diversification. With the volatility of demand, there is a dynamic volatility of work states at the operational level. In order to modify the system with the current parameters determining the variability, it was decided to develop a model based on the rules of Bayesian inference.

The developed model uses the case of Bayesian inference, in which both the a priori distribution and the feature modifying the initial distribution are discrete probability distributions. The a priori distribution was the number of items of a given component from the single order, determined by the empirical distribution based on historical data. It was assumed that the feature modifying the à priori distribution comes from the family of Poisson distributions, where _i_ is the conditional distribution [62]. This means that the value of the probability of occurrence of a single value of the feature modifying the prior distribution is a combination of the products sum of the probability values which is consistent with the Poisson distribution with the determined parameter λ and the corresponding probability value *p_i_* of the occurrence of the parameter λ. Then, the feature takes the form of a conditional distribution with a fixed parameter λ. Furthermore, λ*_i_* is a random variable defined as follows:

(14) $$\forall \;i=1,2,\ldots ,n:\;P\left(\lambda =\lambda_{i}\right)=p_{i}\;\wedge \;\overset{n}{\underset{i=1}{\sum }}p_{i}=1,\;p_{i}\geq 0$$

The system in which the feature assumes a certain value of any distribution for the family of established distributions determines the development of a dynamic model using conditional probability. For this reason, the conditional distribution of the feature with a fixed parameter λ equal to *λ_i_* is a discrete distribution of the random variable:

(15) $$X|\lambda =\lambda_{i}\sim Poisson\left(\lambda_{i}\right)$$

where the distribution of the feature is defined as follows:

(16) $$\forall \;k=0,1,2,\ldots :\;P\left(X=k\right)=\overset{n}{\underset{i=1}{\sum }}P\left(X=k|\lambda =\lambda_{i}\right)\cdot P\left(\lambda =\lambda_{i}\right)$$

Moreover, the variable X is a discrete mixed *Poissona* distribution denoted as:

(17) X~MPoisson(λ)

for λ–defined according to the distribution given by the Formula (14). 

According to this assumption, the predictive posterior distribution is determined according to the Formula (18):

(18) $$\Pi_{1}\left(\lambda =\lambda_{i}\right)=\frac{P_{\lambda_{i}}\left(X=k\right)\cdot \Pi_{0}\left(\lambda =\lambda_{i}\right)}{\sum_{i=1}^{n}P_{\lambda_{i}}\left(X=k\right)\cdot \Pi_{0}\left(\lambda =\lambda_{i}\right)},$$

where: $P_{\lambda_{i}}\left(X=k\right)\sim Poisson\left(\lambda_{i}\right)$, while the *X* characteristic modifying the distribution à priori has a fixed *k*-value derived from Poisson’s distribution at a fixed $\lambda_{i}$.

While:

$\Pi_{1}\left(\lambda =\lambda_{i}\right)$—a posteriori predictive discrete probability distribution;
$\Pi_{0}\left(\lambda =\lambda_{i}\right)$—a priori discrete probability distribution;
$P_{\lambda_{i}}\left(X=k\right)$—the feature distribution for the fixed $\lambda_{i}$, which comes from the family of Poisson distributions.

If the a priori discrete probability distribution a priori $\Pi_{0}\left(\lambda =\lambda_{i}\right)=P_{i}$ and the density function of the feature distribution follows the Poisson distribution $P_{\lambda_{i}}\left(X=k\right)\sim Poisson\left(\lambda_{i}\right)$, determined by the formula:

(19) $$P_{\lambda_{i}}\left(X=k\right)=\frac{\lambda_{i}{}^{k}\cdot e^{-\lambda_{i}}}{k!}$$

then after substituting to Formula (18) and performing transformations, we finally obtain a posterior predictive probability distribution $\Pi_{1}\left(\lambda =\lambda_{i}\right)$, where ∀ i=1,2,…,n:

- for a system sampled with a single value of characteristic X=k,
  (20) $$\Pi_{1}\left(\lambda =\lambda_{i}\right)=\frac{\frac{\lambda_{i}{}^{k}\cdot e^{-\lambda_{i}}}{k!}\cdot P_{i}}{\sum_{i=1}^{n}\frac{\lambda_{i}{}^{k}\cdot e^{-\lambda_{i}}}{k!}\cdot P_{i}},$$
- for a system sampled with several values of characteristic $X=k_{1},k_{2},\ldots ,k_{l}$
  (21) $$\Pi_{1}\left(\lambda =\lambda_{i}\right)=\frac{{\left(e^{-\lambda_{i}}\right)}^{l}\cdot P_{i}\prod_{j=1}^{l}\frac{\lambda_{i}{}^{k_{j}}}{k_{j}!}}{\sum_{i=1}^{n}{\left(e^{-\lambda_{i}}\right)}^{l}\cdot P_{i}\prod_{j=1}^{l}\frac{\lambda_{i}{}^{k_{j}}}{k_{j}!}},$$
  where: $P_{i}$—a priori probability, k—value of a single-sampled feature; $k_{j}$—the value of a feature sampled several times for j=1,…,l; $\lambda_{i}$—the parameter of the Poisson distribution, which is a random variable X|λ defined by a Formula (16).

The developed algorithm takes into account the rules of Bayesian inference that enable predictive determination of the values of the variability indices defined by the Equations (20) and (21). This is a key novelty in the proposed research approach. Ultimately, this algorithm is to be used to balance the distribution of tasks among individual production stations in such a way as to obtain the expected stability of the system’s operating states. The current system did not guarantee this, which resulted in certain losses. By controlling the value of the variability index, it will be possible to reduce the waste from the *mura* group in the production scheduling process. This is a significant benefit for the examined enterprise. 

## 4. Results

The developed algorithm was validated in a separate subsystem. The master system, which is also a separate department from the entire production structure, consists of twenty-one different machine park (Figure 1). Moreover, due to technological and organisational reasons for the analysed structure, the system was divided into two separate sets. Set 1 is the centre of eight machines, while set 2 is nine objects (machines). After the analysis of the continuity of material flow and the sequence of implementation of technological processes, it was decided to adopt a system of nine machines with three different production cells for further analyses.

**Figure 5:**
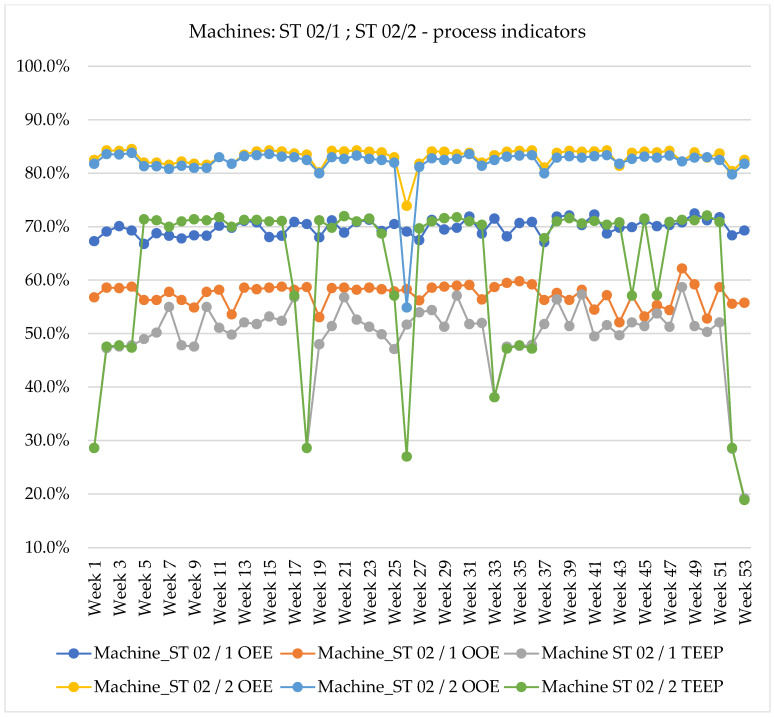
Overall Equipment Effectiveness (OEE), Overall Operations Effectiveness (OOE), Total Effective Equipment Performance (TEEP) indicators for machines ST 02/1 and ST 02/2.

The presented results show changes in the values of the OEE, OOE, and TEEP indicators. It can be observed that the average overall machine efficiency (OEE) is around 80% over the entire period for four plants. For individual machines, the average OEE values are as follows: SL 07/14–79.8%; SK 04/2–81.1%; SK 04/1–83.1%; ST 02/2–83.1%. Only the average value of the total efficiency of the ST 02/1 machine was 69.8%. The characteristic level of OEE recorded in industry is between 65% and 80% (most manufacturing companies are characterised by OEE scores closer to 60%) [64]. The level of 85% is considered to be “world class enterprises” [65,66]. The values of the obtained indicators in the described case should be considered very good. It should be emphasized, however, that the analysed system is aimed at maintaining a high level of quality and all times of task completion are determined in such a way as not to force haste during their implementation. Each planned inspection time and the planned time of machine changeover are approved by the machine operator. Occasionally there are situations in which the machine utilisation drops (P) or the planned changeover time is exceeded. The only losses of OEE indicators result from machine failures—e.g., week 26 in Figure 5.

In order to improve production efficiency, the OOE indicator is used in parallel with the OEE indicator. The OOE indicator conditions the availability from the operating time of the machine. In many guides and publications, OOE is referred to as the global Overall Effectiveness of Equipment (OEE_G_) [67]. Then the earlier indicator is referred to as the technical Overall Effectiveness of Equipment (OEE_T_). The average values of the OOE indicators for the analysed objects are as follows: SL 07/14–76.4%; SK 04/2–71.5%; SK 04/1–61.9%; ST 02/2–81.9.0%; ST 02/1–57.3%.

The global assessment of the effectiveness of the use of the machinery park is the application of the TEEP indicator. The indicator in relation to the previous two (OEE and OOE) takes into account the largest number of loss areas. In determining the value of the TEEP indicator, the component of total availability is considered. In any enterprise it is impossible to achieve values of TEEP equal to 100%. Such value of the indicator means that the company does not experience any downtime (planned and unplanned), speed drops and production of defects; it would be a state of maximum machine utilisation. For a separate system of five machines, the average values of the TEEP indicator are as follows: SL 07/14–62.1%; SK 04/2–61.6%; SK 04/1–53.6%; ST 02/2–62.4%; ST 02/1–49.4%.

Table 1 presents the values of the OEE, OOE, and TEEP indicators for the analysed objects. Due to the large variety and variability of production orders, each machine is defined by a personalized standard value.

Improving processes through the implementation of lean production solutions, which are used in industries with serial or mass production, results in a decrease in quality in the analysed class of production systems. Bad quality determines higher costs for production, which is characterised by a high unit cost anyway. The implementation of lean production solutions is irrational in production systems where the main factor of work efficiency is perfect quality, not machine efficiency. Improvement of production processes in the analysed system, as in other enterprises, focuses on increasing the use of real production time. However, managers are fully aware that the main advantage of the company is its flexibility and full adjustment to customers’ expectations. For this reason, it was necessary to develop a system for controlling the variability of the system operating states.

**Figure 6:**
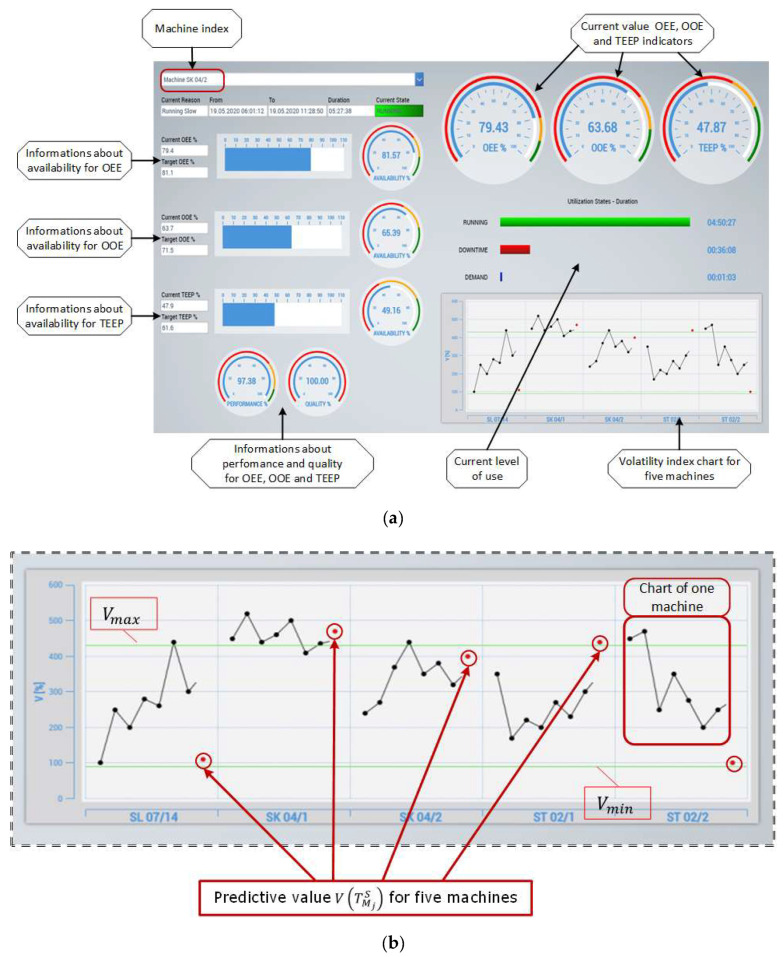
(**a**). The screenshot of the proprietary Manufacturing Executions System (MES) application. (**b**). The screenshot of volatility index chart.

As a result of the application of the algorithms presented above, a real-time data presentation system was developed, which relates to the key values of the parameters of the separated production area. Figure 6a,b show a screenshot of the proprietary MES application. For five physically separated objects, values of the OEE, OOE, TEEP, and *V* indicators in real time can be controlled. The scope of data presentation can be freely specified depending on the information needs of the decision-makers. However, after starting the application, data for the current change from the beginning of its duration is presented. In the lower right corner, there is a graph of the volatility index *V* determined by the Equation (4). The black points show the values of indicators for the already assigned set of tasks. The red points are the predictive values of the *V* index determined on the basis of the planned production schedule, taking into account the rules of Bayesian inference.

**Figure 7:**
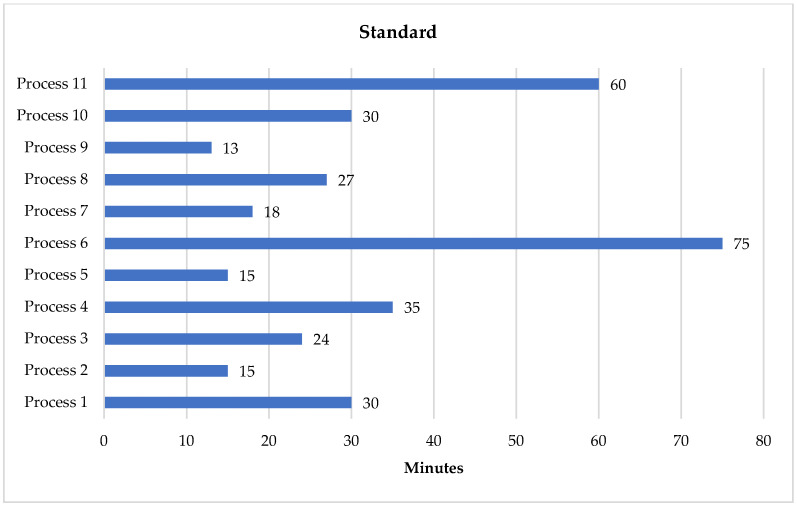
The standard implementation times for subsequent processes.

For the analysed processes, the values of the variability indices were calculated in accordance with Formula (6). Due to the large diversification of the production assortment, the product family was separated, which showed the highest repeatability of orders. Figure 7 shows standard implementation times for subsequent processes, which the company determined based on the results obtained in 2019. Table 2 presents the values of the obtained parameters, which were recorded after the implementation of the developed solution. The comparison of the obtained results with the applicable performance standards revealed that not all processes meet the established requirements. The analysis of parameters allowed to conclude that the product quality control process has the greatest impact on the generation of variability in the system. This is due to the fact that for each production batch a comprehensive inspection of the first element is mandatory. The internal audit team is responsible for carrying out this process. After accepting the quality of workmanship, the machine is started in order to implement the remaining items from the order. This procedure is used to minimise the costs associated with the manufacture of incorrect (defective) pieces. One of the key factors causing a decrease in productivity is therefore the control of product performance.

The analysis of the separated product family concerned the assortment, the technological production stages of which can be grouped in 11 subsequent processes. It should be noted that individual processes are carried out by CNC machine parks with the use of different cutting tools. Therefore, each individual process (1 through 11) is a collection of production steps. For example, in the defined ‘Process 1’, three technologically separate milling, rough turning and drilling processes are performed. A similar situation of grouping technologically separate processes occurs in the following, defined as: ‘Process 3’, ‘Process 4’, ‘Process 6’, ‘Process 7’, ‘Process 8’, and ‘Process 10’. ‘Process 2’, ‘Process 5’, ‘Process 9’, and ‘Process 11’ are stages of control. Compliance checks are carried out by machine operators for Processes 2, 5, and 9, while the QCD performs control as part of ‘Process 11’. In order to identify possible aviation defects, it is necessary to provide detailed information about the product. For this reason, the semi-finished product is included into the catalogue with an individual index number assignment if its quality tests meet the requirements. For each index number, the databases collect information on: production batch number, data of the operator producing the product, data of persons performing quality checks, and all the results of each measurement control carried out.

The human factor plays a large part in the quality control processes. For this reason, the greatest differences in implementation times occur in the following processes: ‘Process 2’, ‘Process 5’, ‘Process 9’—variability above 200%. For ‘Process 11’, the variability in relation to the expected value of the system is 28%—this is the result of the execution time standard well estimated by the technologist. Moreover, it should be noted that this is the last step in the flow of the manufactured component. Sending a non-conforming product to the customer is disproportionately expensive, therefore the process control time standard in ‘Process 11’ is four times longer than that of Processes 2, 5, and 9. Table 2 presents the results of the tests for the algorithm presented in the article.

The surveyed company is constantly testing a number of solutions to improve the process flow and product verification techniques. Most often, managers’ attention was focused on minimizing the operator’s waiting time for verification results. During the validation period of the developed algorithm, it was decided to test the solution with prioritization. Lower and upper control lines were introduced for the values of the volatility index V, which relate to the first and third quartiles of empirical values from a given range. The highest priority was given to those semi-finished products whose *V* value maximally exceeded the third quartile. Defining the priority value in relation to the third quartile resulted from a simple rule of minimising the waiting time for the results of the verification of the correctness of execution. Table 3 presents the values of the variability index determined according to Equation (6) after introducing the priority of performance verification control. The collected data were compared with the values of the variability index obtained before assigning the priority. As a result of the introduced change, a significant reduction in the spread of data was obtained in most cases, which is confirmed by the difference between the values of both variants.

Thanks to the implemented solution, it is possible to control the variability in the adopted production system on an ongoing basis. The considered system is a flexible system which is confirmed by relatively high values of the volatility index. For the assumed case, the values of variability ranging from 40% to almost 300% were obtained. Bearing in mind that the considerations did not take into account all machines and all material flow streams, it is concluded that for the assumed case this variability is relatively low.

## 5. Discussion and Conclusions

Due to the growing popularity of the Industry 4.0 concept, enterprises are looking for solutions that will allow them to automate selected processes in the organisation. Automation usually concerns repetitive processes, in particular functions related to monitoring, reporting, and providing relevant information supporting the decision-making processes of managers. Data exchange in Industry 4.0 concerns not only the communication of machines with each other, but also between the machine and the human being.

Industry 4.0 has created a new type of competition between enterprises. This prompts companies to seek fundamental solutions in quality, performance and agility in their processes and products, hence the manufacturing execution system (MES) has been indispensable to assist them in this process [68]. The system supports the production process, providing access to information displayed in graphics that enables monitoring and analysis of production, as well as products and equipment status, making it possible to trace its use which leads to idleness minimization. The use of MES systems is also justified in the context of the increasingly popular Lean Manufacturing strategy, which is also often used in the aviation sector. Several authors state in their research that the MES fills a gap in the integration between Industry 4.0 and Lean Manufacturing, i.e., by implementing Industry 4.0, companies will become lean because of the need to have well-defined processes [8,69]. For this reason, a new solution was proposed for a selected class of production systems with a high level of automation, which are characterised by flexibility and agility at the operational level. This class of systems is characterised by the production of a wide and varied range of products, which forced the developed optimisation algorithm to take into account the dynamic variability of system operating states. The algorithm also uses the rules of Bayesian inference, which enable multiple adjustments of the model to the existing environmental conditions, without the need to formulate a new description of reality.

The model presented in Section 3 was implemented at selected stations in a separate production cell. The presented concept is an individual approach to assessing the effectiveness of the system. The greatest challenge in the conducted research was the collection of reliable measurement data. CNC machines are used in the production system under consideration, and it is difficult to install additional software from the user level to enable data collection. Installation of additional software requires a specialised authorised service. Many manufacturers prevent the installation of MES software, thus forcing a production company to purchase their own solutions. In this case, the problem was more complex as the proprietary software was very different from the one generally available on the market. Moreover, these were its first tests on a real object. Considering that any new solution during testing reveals unforeseen errors that need to be corrected, then the intervention of specialists would be necessary each time. Therefore, during the validation it was decided to collect information from the Andon system signals. Each change of the impulse blocked the machine and the operator was obliged to classify the reason for the stoppage. This activity results from the necessity to obtain detailed data on various operating states of the machine. The Andon system most often classifies three states: green—working, yellow—waiting, red—failure. In the case under consideration, the following states were taken into account: operation—green, failure—red, standstill—yellow, which can be caused by two variants: 1. quality control—blue, 2. Changeover—purple. Reliable classification of the stoppages made by the operator was particularly important for the proper verification of the developed solution.

The application of the presented solution in a real facility allowed to determine the production areas which are the determinant of system instability. In the presented example it is the Process 11. Despite the fact that in Table 2 Process 11 reaches *V* = 28% (the least of all), in systemic terms this process determines the greatest variability of the system. Process 11 is carried out in a dedicated quality control cell and is key to closing the production order. Without its execution, the order appears in the system as unfinished. This department operates according to internal procedures, which quite often are not correlated with the material flows in the production area. For this reason, the pioneering tactic of prioritising the order of the control process was tested for the presented solution. As a result, for the considered material flows, the cumulative time for the implementation of all tasks in the area of the eleven processes was shortened, which translated into a 59% reduction in instability in the entire system under consideration. Therefore, it should be concluded that for the separated system, the correctness of the implementation of the presented algorithm—which takes into account the current control of the variability—has been confirmed.

The implementation of the developed algorithm allowed to control the generated variability in real time. The developed solutions were verified for a separate system of five machines. The article presents the results obtained for a separate product family, which shows relative stability and repeatability of demand. The deliberate introduction of limiting the area of analyses resulted from the verification of the validity of the assumptions and the necessity to determine the variability. In the next stages of the development of the presented algorithm, methods of optimising the *V_min_* and *V_max_* levels will be developed. It is also becoming extremely important to conduct research related to the optimisation of task scheduling for nest production structures showing a high level of flow complexity. These aspects will be the subject of further research conducted by the authors of the article.

## Figures and Tables

**Table 1:** The values of OEE, OOE, and TEEP indicators for the analyzed objects.

Indicator	Parameter	SL 07/14	SK 04/2	SK 04/1	ST 02/2	ST 02/1
OEE	Minimum value [%]	63.1	79.1	80.7	73.9	66.8
	Maximum value [%]	81.6	82.1	83.9	84.5	72.5
	Median [%]	80.2	81.3	83.2	83.8	69.9
	Number of weeks with a reduced indicator’s value	8	17	19	20	23
	Average level of decrease in the indicator’s value [%]	2.7	0.7	0.6	1.6	1.4
OOE	Minimum value [%]	56.9	55.4	45.4	54.9	52.1
	Maximum value [%]	80.8	73.4	64.2	83.8	62.2
	Median [%]	77.9	72.5	62.9	82.7	58.2
	Number of weeks with a reduced indicator’s value	8	10	9	16	21
	Average level of decrease in the indicator’s value [%]	8.1	4.5	5.1	2.5	2.1
TEEP	Minimum value [%]	18.3	18.7	19.1	18.9	19.2
	Maximum value [%]	72.1	71.6	61.3	72.1	58.7
	Median [%]	71.0	69.1	57.2	70.9	51.4
	Number of weeks with a reduced indicator’s value	18	18	17	16	16
	Average level of decrease in the indicator’s value [%]	17.1	15.9	10.8	19.7	7.6

**Table 2:** The values of the parameters obtained after implementing the developed solution.

Number Process	Max [min]	Min [min]	Average [min]	Median [min]	Standard Deviation [min]	Volatility Index [%]
Process 1	90	30	51	45	21:11	74
Process 2	15	10	13.3	14	1:37	284
Process 3	26	24	24.7	24.5	0:47	153
Process 4	38	32	34.9	35	1:49	108
Process 5	26	13	17	17.5	2:08	222
Process 6	120	70	95.7	96	13:20	40
Process 7	24	17	19	18.5	2:06	199
Process 8	29	15	27.1	27	1:27	140
Process 9	15	11	13	12.5	1:44	291
Process 10	39	31	36.4	37	2:20	104
Process 11	150	100	133	140	16:10	28

**Table 3:** Comparison of the volatility index before and after prioritization.

Number Process	Volatility Index BEFORE Priority Introduction [%]	Volatility Index AFTER Priority Introduction [%]	Difference BEFORE-AFTER [%]
Process 1	74	43	31
Process 2	284	165	119
Process 3	153	89	64
Process 4	108	63	45
Process 5	222	129	93
Process 6	40	25	15
Process 7	199	155	84
Process 8	140	81	59
Process 9	291	168	123
Process 10	104	60	44
Process 11	28	61	-33
